# Biohydrogen Production and Kinetic Modeling Using Sediment Microorganisms of Pichavaram Mangroves, India

**DOI:** 10.1155/2013/265618

**Published:** 2013-11-11

**Authors:** P. Mullai, Eldon R. Rene, K. Sridevi

**Affiliations:** ^1^Pollution Control Research Laboratory, Department of Chemical Engineering, Annamalai University, Annamalai Nagar, Tamil Nadu 608 002, India; ^2^Core Group Pollution Prevention and Resource Recovery, Department of Environmental Engineering and Water Technology, UNESCO-IHE, P.O. Box 3015, 2601 DA, Delft, The Netherlands

## Abstract

Mangrove sediments host rich assemblages of microorganisms, predominantly mixed bacterial cultures, which can be efficiently used for biohydrogen production through anaerobic dark fermentation. The influence of process parameters such as effect of initial glucose concentration, initial medium pH, and trace metal (Fe^2+^) concentration was investigated in this study. A maximum hydrogen yield of 2.34, 2.3, and 2.6 mol H_2_ mol^−1^ glucose, respectively, was obtained under the following set of optimal conditions: initial substrate concentration—10,000 mg L^−1^, initial pH—6.0, and ferrous sulphate concentration—100 mg L^−1^, respectively. The addition of trace metal to the medium (100 mg L^−1^ FeSO_4_ *·*7H_2_O) enhanced the biohydrogen yield from 2.3 mol H_2_ mol^−1^ glucose to 2.6 mol H_2_ mol^−1^ glucose. Furthermore, the experimental data was subjected to kinetic analysis and the kinetic constants were estimated with the help of well-known kinetic models available in the literature, namely, Monod model, logistic model and Luedeking-Piret model. The model fitting was found to be in good agreement with the experimental observations, for all the models, with regression coefficient values >0.92.

## 1. Introduction

Fossil Fuels are the primary energy source for the world's increasing energy consumption. According to a recent survey, total world energy use rises from 524 quadrillion British thermal units (Btu) in 2010 to 630 quadrillion Btu in 2020 and to 820 quadrillion Btu in 2040 [1]. This fossil fuel eventually leads to foreseeable depletion due to limited energy resources; however, in the last few years, research and development activities pertaining to large-scale production of alternate resources of energy such as biodiesel, biohydrogen and bioethanol have risen [2–8]. In the days of fast depleting fossil fuel, biohydrogen has become a promising and viable energy source owing to its inherent advantages: zero-pollution, carbon-free, inexhaustible, recyclable, and highest energy density. However, most of hydrogen is currently produced from non-renewable sources using natural gas (50%), petroleum-derived naphthenes and distillates (30%), coal (18%), and electricity produced from variety of fuels (2%). Since this strategy leads to the depletion of non-renewable energy sources and is considered as a less ecofriendly alternative, it becomes crucial to go in for the production of sustainable energy source.

Biohydrogen production through anaerobic fermentation is a sustainable alternate for the energy crisis and green environment [9–12]. Fermentative hydrogen production processes are technically feasible and economically competitive and have large-scale commercialization possibilities [8, 13–16]. The present work focuses on biohydrogen production by dark fermentative approach using mangrove sediments of Pichavaram (located in Tamil Nadu, India). It is known that no research has been made using the sediments of mangroves, new mixed consortia to produce biohydrogen. Mangrove sediments are inherently rich in organic content [17–19]. The advantages of this sediment can be summarized as follows: flexible substrate utilization and the simplicity of handling, no major storage problems, no problems with strain degradation, no preculturing required, and sediments are available at low cost.

A kinetic model can adequately describe the relationship among the different state variables and explain the behavior of fermentation quantitatively by providing useful information that can be subsequently used for analysis, design, and operation of any fermentation process [20–22]. The unstructured kinetic models are frequently employed for modeling microbial systems because they are simple, yet can provide useful information about the process [11, 23, 24]. In this study, three unstructured kinetic models, namely, Monod, logistic, and Luedeking-Piret models [25, 26] were used to determine the kinetic parameters.

## 2. Materials and Methods

### 2.1. Selective Enrichment on Biohydrogen Producing Mangrove Sediments

The sediments were collected from the mangrove rhizosphere of Pichavaram, Tamil Nadu, India, at a depth of 100 cm, and later stored in sterile polythene bags. Heat-shock treatment was done on this sediment sample, by constant heating at 110°C for 2 h, in order to stimulate spore germination and eliminate all vegetative cells, particularly methanogens. The coarse particles were removed using a stainless steel mesh, while the finer fractions were stored at 4°C [27].

### 2.2. Nutrient Medium

The nutrient medium (non-sterilized) used in this study had the following chemical composition (per litre): NH_4_Cl—0.5 mg, K_2_HPO_4_—0.25 mg, MgCl_2_·6H_2_O—0.3 mg, NiSO_4_—0.016 mg, CoCl_2_—0.025 mg, ZnCl_2_—0.0115 mg, CuCl_2_—0.0105 mg, CaCl_2_—0.005 mg, and MnCl_2_—0.015 mg.

### 2.3. Batch Experiments

Batch tests were conducted in duplicate, in 1 L Erlenmeyer flasks (working volume: 0.7 L), fitted air-tightly with rubber septum, and adequately sealed using commercially available fix gels. The effect of process parameters on biohydrogen yield, namely, the influence of initial substrate concentration (glucose), initial pH, and trace metal, Fe^2+^ concentration, was evaluated by carrying out experiments at different low to high levels of these parameters, and the average values of biohydrogen yield were presented. The pH of the growth medium was adjusted using 1N HCl or 1N NaOH during the start of the experiments. The growth medium was inoculated with 100 g of pretreated sediment under aseptic conditions, and the flasks were incubated at 35°C for fermentation.

### 2.4. Analytical Methods

The biohydrogen gas was measured using wet gas flow meter (Toshniwal, India). The gas content was analyzed using a gas chromatograph (Shimadzu, 221-70026-34, Japan) equipped with a thermal conductivity detector (TCD), and the column was packed with dual packed column. The operating temperatures of the column, detector and injector, were 40°C, 80°C, and 50°C, respectively. Biomass concentration was measured as volatile suspended solid (VSS) and analyzed according to Standard Methods [28]. Glucose concentration was measured by DNS method using spectrophotometer (Elico, India) at a *λ*_max⁡_ of 550 nm [29]. The sludge granules were characterized using scanning electron microscope (SEM) (JEOL-JSM, 5300, Japan) at a resolution of 4.5 nm at 15 kVA with a working distance of 8 mm.

## 3. Results and Discussion

Biohydrogen fermentation reached nearly constant values at the end of 120 h for each batch tests, including their duplicates. Glucose degradation efficiencies, cumulative biohydrogen gas, and hydrogen yields were calculated for each set of experimental condition.

### 3.1. Effect of Initial Glucose Concentration

**Figure 1:**
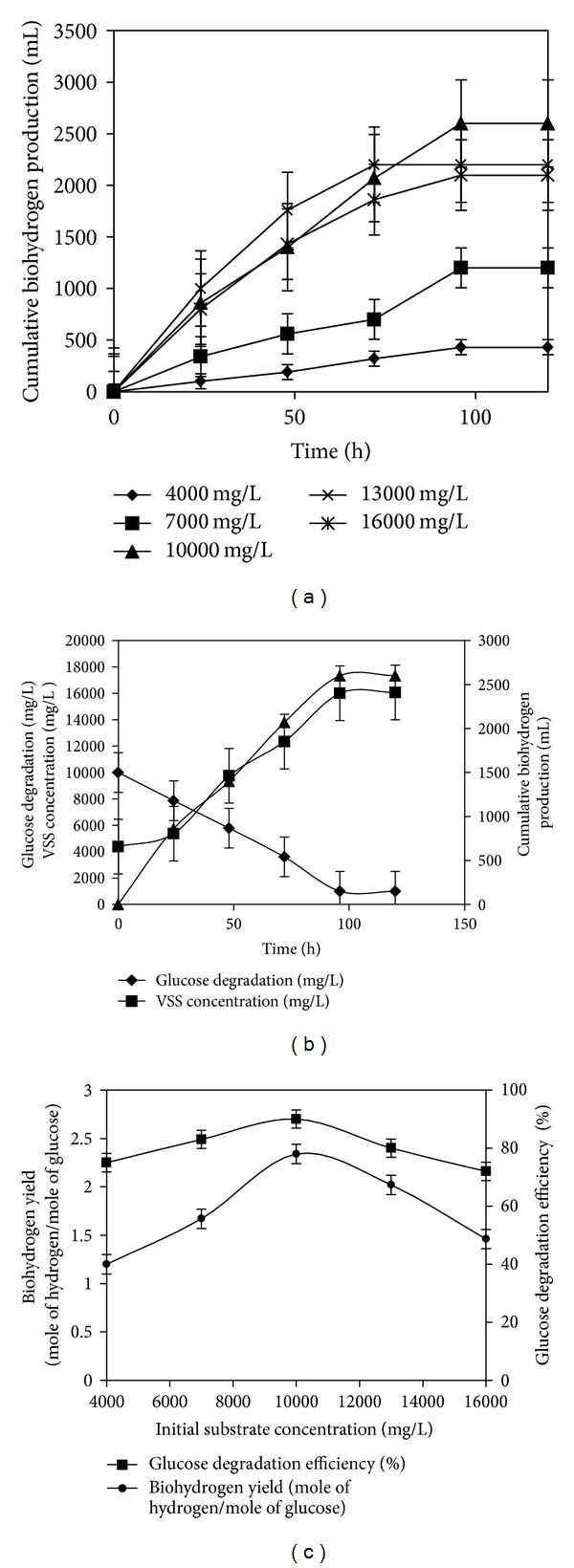
(a) Profile of cumulative biohydrogen production at various initial glucose concentrations. (b) Dynamic profile of glucose degradation, biomass concentration, and cumulative biohydrogen production. (c) Biohydrogen yield and glucose degradation efficiency for various initial glucose concentrations.

For initial glucose concentrations of 4,000, 7,000, 10,000, 13,000, and 16,000 mg L^−1^, the values of cumulative biohydrogen production and glucose degrading efficiencies were 430, 1190, 2600, 2200, and 2099 mL and 75, 83, 90, 80, and 72%, respectively (Figure 1). The effect of initial glucose concentration was observed when the initial medium pH was kept constant at 6.0 for all the test vials. It was observed that biohydrogen production increased with an increase in glucose concentration from 4,000 to 10,000 mg L^−1^, and after that the biohydrogen production decreased with further increase in glucose concentration. A maximum biohydrogen yield of 2.34 mol H_2_ mol^−1^ glucose was obtained when initial glucose concentration was 10,000 mg L^−1^. Furthermore, when initial glucose concentration was increased to 13,000 mg L^−1^ and 16,000 mg L^−1^, the hydrogen yield obtained was 2.02 and 1.46 mol H_2_ mol^−1^ glucose, respectively (Figure 1). The decrease in biohydrogen production at higher substrate concentrations might be due to the formation of more volatile fatty acids (data not shown here) which resulted in over-acidification of bacterial cultures, thereby reducing the medium pH, and thus inhibited fermentation. Several reports have shown that although high substrate concentrations showed high biohydrogen production initially, they tend to drop to low levels due to simultaneous acid inhibition, and increased partial pressure of hydrogen in the flask [30, 31]. Maintaining the carbon source levels at an optimum, in bioreactors, is an important parameter during pilot-scale trials and during the continuous production of biohydrogen. Failure to do so could affect the growth rate of the microorganism, its specific substrate utilization rate, enzyme activity, and overall yield of the process itself. Hence, to avoid the formation of volatile fatty acids and the phenomena of substrate inhibitions, the concentration of the substrate (glucose) in the liquid-phase must be maintained at optimal levels.

### 3.2. Effect of Medium pH

**Figure 2:**
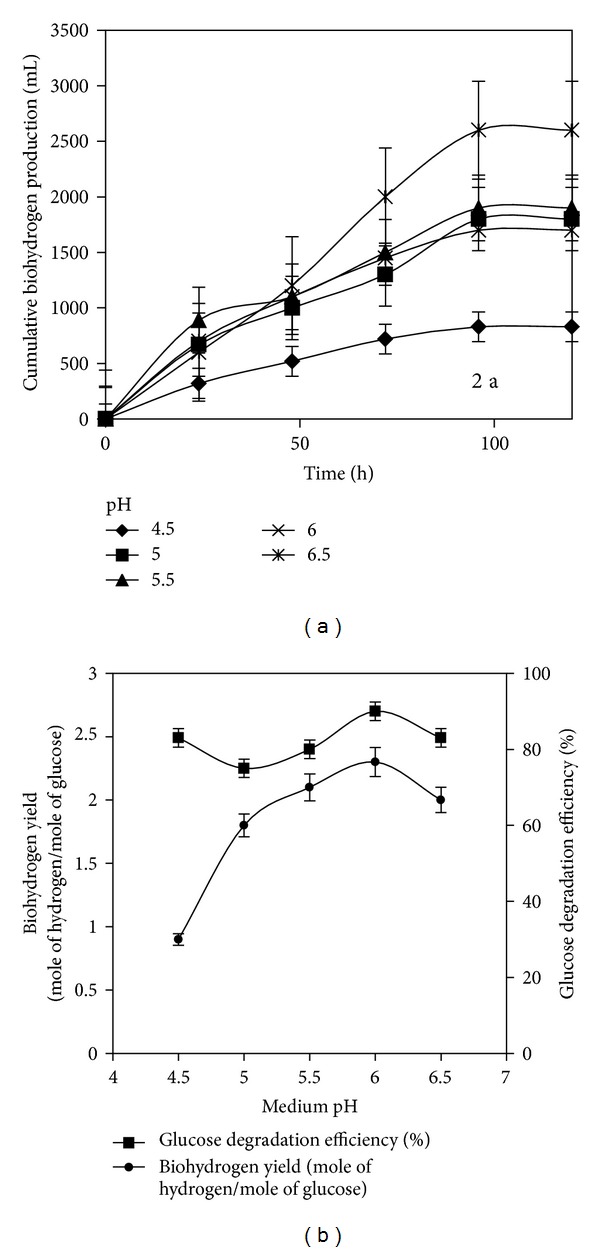
(a) Profile of cumulative biohydrogen production at various medium pH. (b) Biohydrogen yield and glucose degradation efficiency for various medium pH.

The profile of cumulative biohydrogen gas production at various initial medium pH conditions is shown in Figure 2. The optimum initial glucose concentration of 10,000 mg L^−1^ was constantly maintained for these experiments. The substrate degradation efficiencies obtained were 83, 75, 80, 90, and 83%, respectively, at initial pH values of 4.5, 5.0, 5.5, 6.0, and 6.5. The final pH of these test vials at the end of the test period ranged from 1.9 to 3.4. The medium pH is an important operational parameter for hydrogen production, since it affects anaerobic pathways and the activities of hydrogenase enzymes [32]. When the initial medium pH was varied by keeping initial substrate concentration constant at 10,000 mg L^−1^, the maximum hydrogen yield of 2.3 mol H_2_ mol^−1^ glucose was obtained at an initial pH of 6.0 (Figure 2). Initially, when the medium pH was at 4.5, the lowest hydrogen yield of 0.9 mol H_2_ mol^−1^ glucose obtained indicated that the higher acidic condition inhibited the fermentation. The hydrogen yield substantially increased to 2.3 mol H_2_ mol^−1^ glucose at the pH of 6.0. The hydrogen yield decreased to 2.0 mol H_2_ mol^−1^ glucose at a higher pH value (6.5). It was found that, under near neutral pH condition, a significant amount of substrates was consumed by bacterial growth other than hydrogen production which was verified by the higher biomass concentration at higher pH. Thus, it could be stated that the favourable pH for this mixed bacterial culture was 6.0. Similar results of maximum hydrogen production at the pH of 6.0 were reported [33].

### 3.3. Effect of Fe^2+^ Concentration

**Figure 3:**
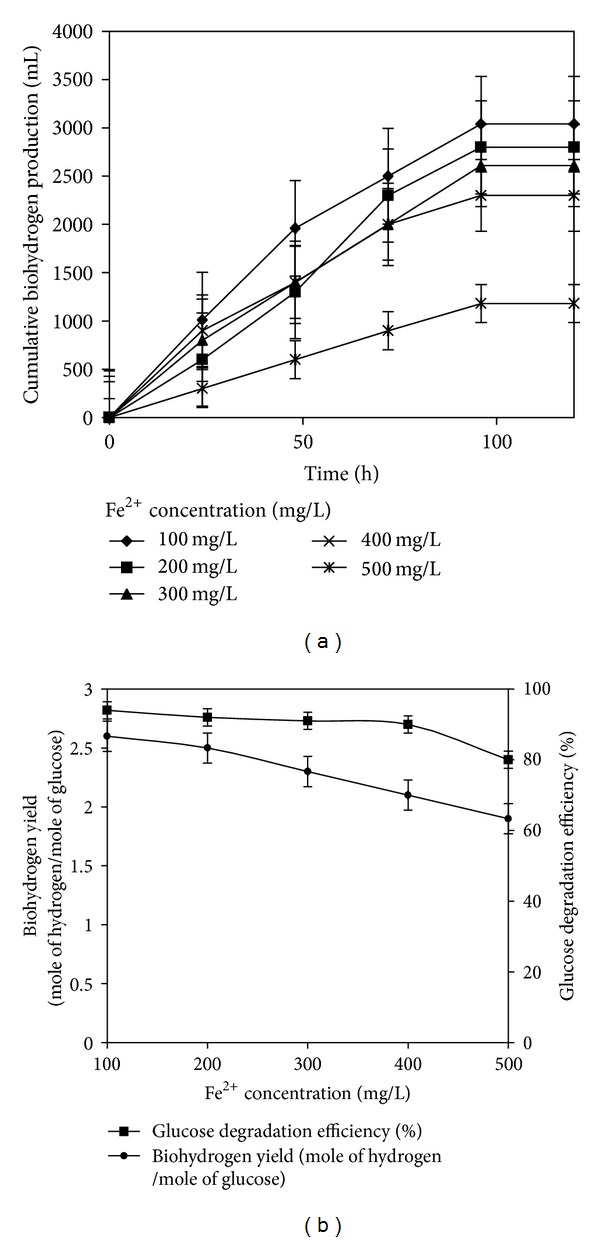
(a) Profile of cumulative biohydrogen production at different Fe^2+^ concentrations. (b) Biohydrogen yield and glucose degradation efficiency for various Fe^2+^ concentrations.

Figure 3 illustrates the effect of fermentation time on the cumulative hydrogen production in batch tests under different Fe^2+^ concentrations. The values of cumulative biohydrogen production for five different Fe^2+^ concentrations: 100, 200, 300, 400, and 500 mg L^−1^ were 3040, 2800, 2610, 2300, and 1180 mL, respectively, and the corresponding substrate degradation efficiencies were 94, 92, 91, 90, and 80%. Hydrogen yields of 2.6, 2.3, 2.1, 1.8, and 0.9 mol H_2_ mol^−1^ glucose were obtained for various concentrations of iron as illustrated in Figure 3. At 100 mg L^−1^ of Fe^2+^ concentration, the biohydrogen production was at its maximum (2.6 mol H_2_ mol^−1^ glucose), and it was found to decrease when the Fe^2+^ concentration was increased (Figure 3). Similar trend was obtained by previous researchers [34–36]. The addition/presence of Fe^2+^ concentration in the fermentation medium could influence the fermentative hydrogen production by influencing the activity of hydrogenase enzyme. The literature reports have shown that metal ions affect the microrganisms involved in hydrogen fermentation, beyond a threshold concentration range, and these effects include the following: decreased hydrogen production rate, an increase in lag-phase time, and formation of soluble microbial products [34].

### 3.4. Kinetics of Biohydrogen Production in Batch Culture

#### 3.4.1. Cell Growth Kinetics as a Function of Substrate

**Figure 4:**
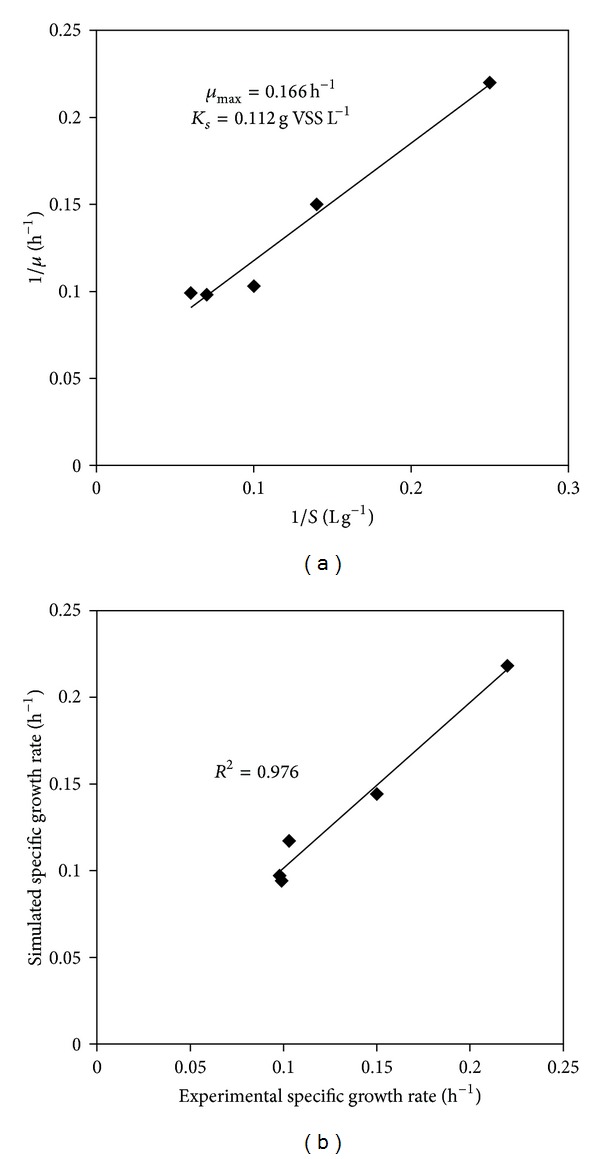
Monod model for substrate utilization kinetics.

Monod kinetics was applied to study the cell growth kinetics during biohydrogen production. Monod kinetics is given by the following equation:

(1) $$\begin{array}{c} \mu =\frac{1}{x}\frac{dx}{dt}=\frac{\mu_{\max}S}{K_{s}+S}, \end{array}$$

where *μ* is the specific growth rate (h^−1^), *μ*_max⁡_ is the maximum specific growth rate (h^−1^), *x* is the cell concentration (g L^−1^), and *K*_*s*_ is the substrate consumption rate constant (g L^−1^). Equation (1) may be linearized, as shown in (2) to estimate the kinetic parameters, and regression analysis is used to find the best fit for a straight line on a plot of 1/*μ* versus 1/*S* to determine the values of *μ*_max⁡_ and *K*_*s*_ (Figure 4):

(2) $$\begin{array}{c} \frac{1}{\mu }=\frac{K_{s}}{\mu_{\max}}\cdot \frac{1}{S}+\frac{1}{\mu_{\max}}. \end{array}$$

Table 1 shows the different values of kinetic parameters obtained from Monod model, while Figure 4 shows the correlation between the model fitted and experimental values. The *μ*_max⁡_ and *K*_*s*_ were calculated as 0.166 h^−1^ and 0.112 g L^−1^ respectively.

#### 3.4.2. Cell Growth Rate as a Function of Cell Concentration

The specific growth rate for the logistic curve relates the change of specific growth rate with respect to change in cell concentration (*x*). The Riccatti equation is given by the following equation:

(3) $$\begin{array}{c} \frac{dx}{dt}=kx\left(1-\beta x\right), \end{array}$$

where *β* = 1/*x*_max⁡_.

On integrating and applying the limits,

(4) $$\begin{array}{c} \int_{x_{0}}^{x}\frac{dx}{x\left(1-\beta x\right)}=k\int_{0}^{t}dt,\\ e^{kt}=\frac{x\left(1-\beta x_{0}\right)}{x_{0}\left(1-\beta x\right)}. \end{array}$$

Rearranging the above equation, cell concentration *x* is given by

(5) $$\begin{array}{c} x=\frac{x_{0}e^{kt}}{1-\beta x_{0}\left(1-e^{kt}\right)}. \end{array}$$

*x*_max⁡_ and *k* kinetic parameters are calculated using logistic curve.

**Figure 5:**
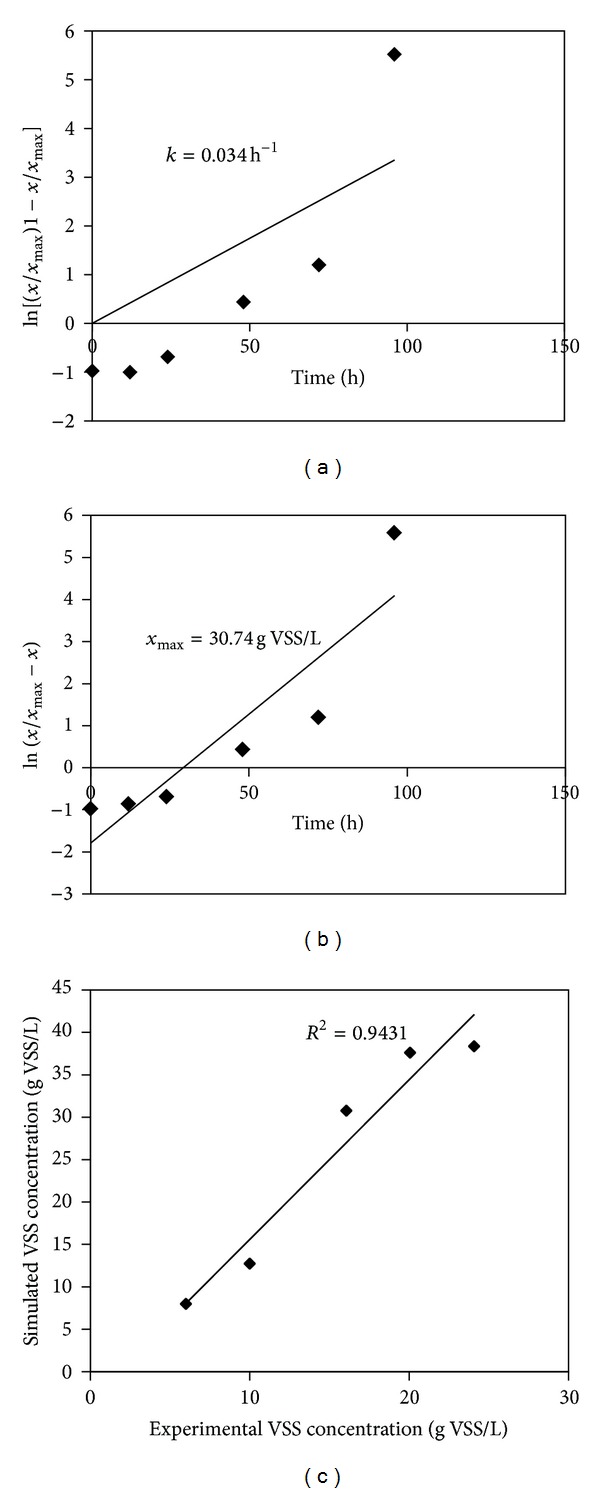
Logistic model for cell growth kinetics.

However, for the purposes of batch hydrogen production experiments, where the initial substrate concentrations and the inoculation volume are kept constant, the logistic model is only a fair approximation of the growth curve. From Figure 5, kinetic parameters were estimated and their values were as follows: *k* = 0.061 h^−1^; *x*_max⁡_ = 30.74 gVSS L^−1^.  Table 2 shows the comparison of different kinetic parameters for the logistic model. The experimental and model fitted specific growth rates were significant with high regression coefficient values. From Figure 5, it could be inferred that the model performed well during the simulation of batch reactors performance, with respect to the glucose and biomass concentration.

#### 3.4.3. Cell Growth Rate as a Function of Product Formation

The Luedeking-Piret model shown in (6) has been widely used to describe the relationship between hydrogen producing bacterial growth rate and product formation rate:

(6) $$\begin{array}{c} \frac{dp}{dt}=Y_{P/x}\frac{dx}{dt}+\beta x, \end{array}$$

where *dp*/*dt* is the product formation rate (h^−1^), *dx*/*dt* is the specific growth rate (h^−1^), *P* is the product (biohydrogen production), *x* is the cell concentration (g L^−1^), *Y*_*P*/*x*_ is the growth associate product yield coefficient, and *β* is the non-growth associated product yield coefficient.

**Figure 6:**
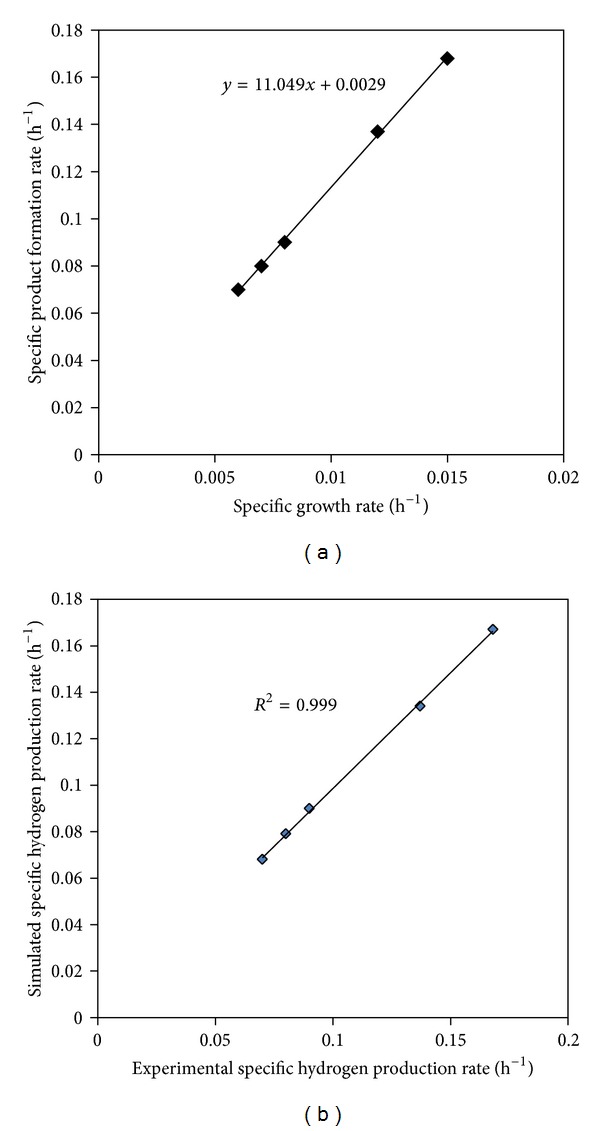
Luedeking-Piret model for product formation kinetics.

Table 3 shows the values of different kinetic parameters estimated for this model. A plot of specific growth rate versus product formation rate, as shown in Figure 6, indicates that hydrogen is purely a growth associated product. The growth associate product yield coefficient (*Y*_*P*/*x*_) was calculated by plotting specific hydrogen production rate versus specific growth rate, and the value was found to be 11.04. From Figure 6, it could be inferred that the model performed well with *R* ^2^ value of 0.999.

#### 3.4.4. Microscopic Examination of Hydrogen Producing Granule

**Figure 7:**
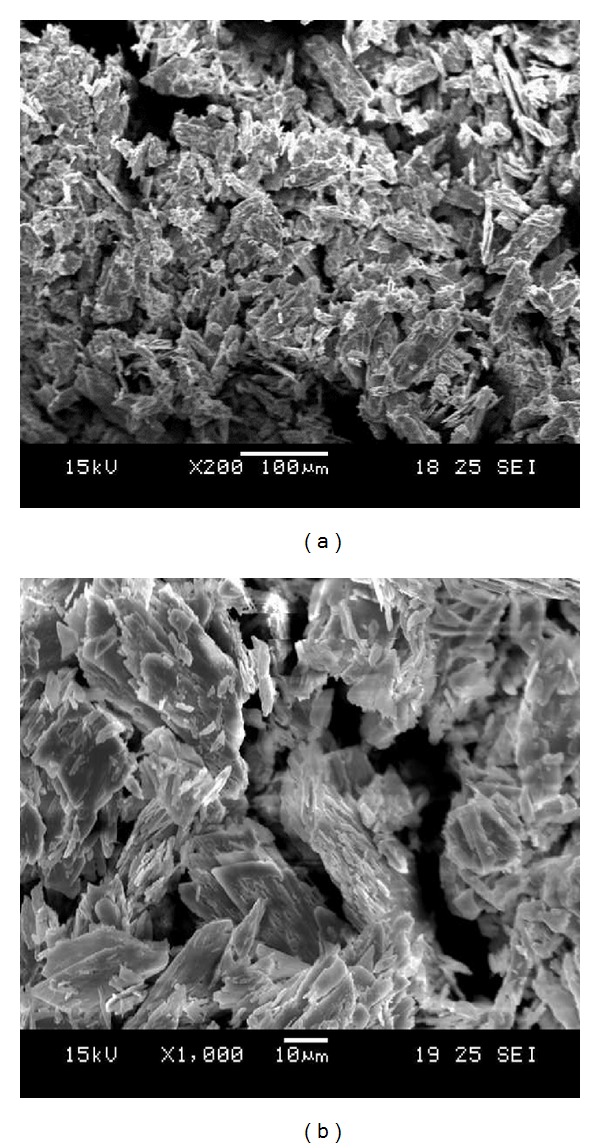
SEM image of typical hydrogen-producing granule.

Scanning electron micrographs showed that the granules had multiple cracks with cavities on the surface (Figure 7). These cavities were likely to facilitate the passage of nutrients and substrate as well as the release of hydrogen. Bacterial cells were distributed all over the granules.

Furthermore, considering the practicality of this research work, microbiological analyses are warranted at this stage to characterize the dominant anaerobic consortium responsible for biohydrogen production. In general, kinetic models are applied in order to study and assess the metabolic features of defined cultures. Further studies in this field should be aimed at the following aspects: optimization studies with different innocula, substrates and process parameters, evaluation of the performance, and economics of a continuous biohydrogen production processes (bioreactors).

## 4. Conclusions

The results from batch tests showed that initial substrate (glucose) concentration, medium pH, and Fe^2+^ concentration had influence on the biohydrogen yield. Maximum biohydrogen yields were found to be 2.34, 2.3, and 2.5 mol H_2_ mol^−1^ glucose at the following conditions: initial substrate concentration—10,000 mg L^−1^, medium pH—6.0, and Fe^2+^ concentration—100 mg L^−1^, respectively. The addition of trace metal to the medium at a concentration of 100 mg L^−1^ was found to enhance biohydrogen production although higher metal ion concentrations reduced biohydrogen production. The kinetics of batch anaerobic hydrogen production was estimated by fitting the experimental data to the well-known unstructured kinetic models. The Monod model, logistic model, and Luedeking-Piret model were used to describe the kinetics of cell growth rate as a function of substrate, cell concentration, and product formation, respectively, in the hydrogen production process, and the corresponding kinetic constants were estimated. The results showed that high regression co-efficient values (*R* ^2^) were obtained between the model fitted and the experimental observations for the different models, namely, as 0.976, 0.943, and 0.999, respectively.

## Figures and Tables

**Table 1:** Comparison of kinetic parameters for Monod model.

Process	Type of culture	Substrate	*µ*_ max_	*K*_*s*_	*R* ^2^	Author
Batch	Mixed anaerobic culture	Sucrose	0.078 h^−1^	—	—	[26]
Batch	*Clostridium pasteurianum* CH4	Sucrose	0.31 h^−1^	4.39 g COD L^−1^	0.935	[37]
Batch	Mixed sludge	Glucose	0.03 g biomass/g biomass/day	—	—	[38]
Batch	Mixed culture	Xylose	0.17 h^−1^	0.75 g/L	—	[39]
Sequential batch	Activated sludge	Glucose	0.125 h^−1^	—	—	[40]
Batch	Acidogenic mixed culture	Glucose	0.163 h^−1^	—	—	[41]
Batch	Acidogenic mixed culture	Fructose	0.108 h^−1^	—	—	[41]
Batch	Anaerobic acclimatized banana stem sludge	Banana stem waste	0.111 h^−1^	0.330 g/L	0.902	[42]
Batch	Sediments of Pichavaram mangroves	Glucose	0.166 h^−1^	0.112 g/L	0.971	Present study

**Table 2:** Comparison of kinetic parameters of logistic model.

Process	Type of culture	Substrate	*k* (h^−1^)	*R* ^2^	Author
Batch	*Rhodobacter sphaeroides *	Malic acid	0.098	0.98	[25]
Batch	Sludge	Glucose	—	0.99	[26]
Batch	Sediments of Pichavaram mangroves	Glucose	0.034	0.943	Present study

**Table 3:** Comparison of kinetic parameters of Luedeking-Piret model.

Process	Type of culture	Substrate	*Y*_*P*/*x*_	*R* ^2^	Author
Batch	*Clostridium butrycum *CGS5	Xylose	0.041	0.910	[37]
Batch	Mixed microflora	Wheat stalk	—	>0.855	[43]
Batch	Sediments of Pichavaram mangroves	Glucose	11.04	0.999	Present study

## Nomenclature

*μ*: Specific growth rate (h^−1^)
Maximum specific growth rate (h^−1^)
*x*: Microbial concentration (g VSS L^−1^)
*x*_0_: Initial microbial concentrations (g VSS L^−1^)
*K*_*s*_: Substrate consumption rate (g L^−1^)
*k*: Apparent specific growth rate (h^−1^)
*x*_max⁡_: Maximum microbial concentration (g VSS L^−1^)
*P*: Cumulative biohydrogen production (mL)
*Y*_*P*/*x*_: Growth associate product yield coefficient
*β*: Non-growth associated product yield coefficient.
